# Human single-chain variable fragment that specifically targets arthritic cartilage

**DOI:** 10.1002/art.27346

**Published:** 2010-04

**Authors:** Chris Hughes, Bjarne Faurholm, Francesco Dell'Accio, Antonio Manzo, Michael Seed, Noha Eltawil, Alessandra Marrelli, David Gould, Christina Subang, Adam Al-Kashi, Cosimo De Bari, Paul Winyard, Yuti Chernajovsky, Ahuva Nissim

**Affiliations:** 1 Barts and The London School of Medicine and Dentistry, Queen Mary University of LondonLondon, UK; 2 University of AberdeenAberdeen, UK; 3 University of ExeterExeter, UK

## Abstract

**Objective:**

To demonstrate that posttranslational modification of type II collagen (CII) by reactive oxygen species (ROS), which are known to be present in inflamed arthritic joints, can give rise to epitopes specific to damaged cartilage in rheumatoid arthritis (RA) and osteoarthritis (OA) and to establish a proof of concept that antibodies specific to ROS-modified CII can be used to target therapeutics specifically to inflamed arthritic joints.

**Methods:**

We used a semisynthetic phage display human antibody library to raise single-chain variable fragments (scFv) specific to ROS-modified CII. The specificity of anti–ROS-modified CII scFv to damaged arthritic cartilage was assessed in vitro by immunostaining articular cartilage from RA and OA patients and from normal controls. The in vivo targeting potential was tested using mice with antigen-induced arthritis, in which localization of anti–ROS-modified CII scFv in the joints was determined. The therapeutic effect of anti–ROS-modified CII scFv fused to soluble murine tumor necrosis factor receptor II–Fc fusion protein (mTNFRII-Fc) was also investigated.

**Results:**

The anti–ROS-modified CII scFv bound to damaged arthritic cartilage from patients with RA and OA but not to normal preserved cartilage. When systemically administered to arthritic mice, the anti–ROS-modified CII accumulated selectively at the inflamed joints. Importantly, when fused to mTNFRII-Fc, it significantly reduced inflammation in arthritic mice, as compared with the effects of mTNFRII-Fc alone or of mTNFRII-Fc fused to an irrelevant scFv.

**Conclusion:**

Our findings indicate that biologic therapeutics can be targeted specifically to arthritic joints and suggest a new approach for the development of novel treatments of arthritis.

Cartilage destruction is a key pathologic feature of joint disorders such as rheumatoid arthritis (RA) and osteoarthritis (OA), conditions that represent a pressing social and economic burden, especially in view of an increasingly aging population. Arthritis is often polyarticular and therefore requires systemic administration of therapeutic agents. Systemic treatment with disease-modifying antirheumatic drugs (DMARDs) is associated with side effects, since such treatment does not deliver pharmacologically active molecules solely to the site of disease activity in the joints.

The problem remains unresolved with biologic DMARDs, including the tumor necrosis factor α (TNFα)–blocking class of proteins, which have been established as a standard in the treatment of RA in patients whose disease has failed to respond to conventional DMARDs (1). However, the financial strain placed on healthcare systems by the prescription of high-priced biologic agents is a major burden (2). In addition, because of the generalized immunosuppression in patients receiving biologic agents, there are safety issues due to the high risk of developing infections (3). Also, a significant number of patients do not respond to anti-TNFα therapy. Therapeutic options for these patients include increasing the dose, switching to an alternative TNF antagonist, or switching to a biologic drug of a different class, such as rituximab, abatacept, (4) and more recently, tocilizumab (5). Regardless of whether TNF, interleukin-6 (IL-6), or CD20 blockade therapy is used, there is an unmet need for the development of novel therapies with improved efficacy and substantially reduced side effects.

In RA, inflammatory cells infiltrate the inflamed synovial membrane (6), producing high levels of inflammatory cytokines, such as TNFα and IL-1 (7), which in turn lead to the production of matrix metalloproteinases (MMPs), which are responsible for the destruction of cartilage (8). Moreover, the influx of infiltrating leukocytes consumes increased amounts of oxygen, resulting in the overproduction of O_2_^**.−**^ radical and leading to the generation of derivative oxidants such as H_2_O_2_, ^.^OH, and HOCl (9–12). An excess of nitric oxide, another key proinflammatory mediator (12), reacts with O_2_^.−^ to form ONOO^−^. Although synovial inflammation in OA is not as extensive as in RA, similar mediators of inflammation are produced either by chondrocytes (13) or by infiltrating B and T lymphocytes (14). As in RA, oxidative stress may also play a major role in the development of OA. Furthermore, the link between OA and aging might be due to excessive levels of reactive oxygen species (ROS) that tip the balance of anabolic and catabolic events, with a resulting loss of homeostasis. Moreover, in OA as well as in RA, cartilage degradation is associated with nonenzymatic glycation, which generates advanced glycation end products (AGEs). A hallmark of AGEs is pentosidine, the levels of which are increased in RA and OA despite the absence of hyperglycemia (15).

We studied the immunopathologic events following ROS-mediated modification of type II collagen (CII), a main and specific component of the cartilage extracellular matrix and a known autoantigen in RA. We have previously reported a substantial increase in binding of RA sera to ROS-modified CII, as compared with binding to native unmodified CII (16). In the current study, assuming that ROS-modified CII is present only in the inflamed joints and using a phage display human antibody library, we identified a human single-chain variable fragment (scFv) that binds specifically to ROS-modified CII. Indeed, the anti–ROS-modified CII scFv, 1-11E, was found to bind specifically to damaged cartilage characteristic of RA and OA, but not normal articular cartilage. Importantly, using a mouse model of monarticular antigen-induced arthritis (AIA), we provide herein a proof of concept that the anti–ROS-modified CII scFv can be used to target therapeutic agents exclusively to damaged cartilage in arthritic joints.

## MATERIALS AND METHODS

### Development of anti–modified CII scFv from the phage display library

CII was prepared from bovine cartilage (17) and subsequently exposed to reactive oxygen–generating systems. Briefly, CII was modified with ^.^OH, HOCl, ONOO^−^, or ribose by overnight incubation at 37°C, as described previously (16). To select for the scFv specific to ROS-modified CII, we used a human semisynthetic scFv library (18). Selection was performed as described previously (online at http://www.geneservice.co.uk/products/proteomic/datasheets/tomlinsonIJ.pdf), with some modifications. To select for phage binding specifically to ROS-modified CII and not to native CII, 3 rounds of subtractive selection were performed using native CII for subtraction. Soluble scFv were obtained from an infected, nonsuppressor *Escherichia coli* HB2151 bacterial strain, as described previously (19). To select for a specific scFv to hen egg lysozyme (HEL), which served as a control, we used a standard protocol.

### Samples of human RA, OA, and normal cartilage

Human RA cartilage samples (provided by Professor C. Montecucco, Università degli Studi di Pavia, Pavia, Italy) were obtained from 2 patients who were diagnosed according to the American College of Rheumatology (formerly, the American Rheumatism Association) revised criteria for RA (20) and who were undergoing total knee joint replacement. At the time of sample collection, the patients were 52 and 47 years old, with a disease duration of 13 and 15 years, respectively. Both patients had been treated with steroids and DMARDs, such as methotrexate and sulfasalazine. OA cartilage samples (also provided by Professor C. Montecucco) were obtained from 3 additional patients undergoing prosthetic knee replacement. Normal cartilage was obtained postmortem and exhibited no histologic evidence of joint pathology. All samples were collected in accordance with institutional ethics policies and regulations (Istituto Ricerca Cura Carattere Scientifico Foundation Policlinico San Matteo, Pavia, Italy).

### Immunohistochemistry

Safranin O staining was performed according to standard protocols (21). For immunostaining, sections 5 μm thick were deparaffinized and hydrated according to standard protocols. After endogenous peroxidase quenching in 3% H_2_O_2_ for 15 minutes, antigen retrieval was performed by incubating slides with 3 mg/ml pepsin (Zymed, Chandlers Ford, UK) for 45 minutes at 37°C. Using a blocking solution of 0.5% bovine serum albumin (BSA) in phosphate buffered saline (PBS), sections were incubated overnight at 4°C with either the selected scFv (10 μg/ml) or control commercial mouse anti-CII antibodies (1:1,000 dilution; Chemicon International, Chandlers Ford, UK). Mouse anti–Myc-Tag antibodies were used to bind to the Myc-Tag incorporated at the carboxy-terminal end of the scFv (1:200 dilution; Santa Cruz Biotechnology, Wembley, UK), which was followed by incubation with a goat anti-mouse biotinylated antibody, using a Vectastain PK-6102 kit according to the instructions of the manufacturer (Vector, Peterborough, UK). Immunostaining with the control commercial mouse antibodies (1:1,000 dilution; Millipore, Watford, UK) was probed with a goat anti-mouse antibody as above. Diaminobenzidine substrate was used as peroxidase substrate (Dako, Ely, UK). Sections were counterstained with hematoxylin and mounted with DPX (BDH, London, UK). Immunohistochemical analysis of the mouse cartilage sections was performed following the same procedure used for the human cartilage specimens, except that a rat anti–Myc-Tag followed by horseradish peroxidase (HRP)–conjugated anti-rat antibodies were used, according to the instructions of the manufacturer (Dako).

### Mouse models of arthritis

We used mice with AIA as a model for inflamed arthritis (22). To obtain an AIA model of chronic arthritis, animals were rechallenged with methylated BSA (mBSA) 1 month after the first challenge with mBSA, which was injected into either inflamed knees or paws as described previously (23). C57BL/6 mice subjected to knee injury were next used as a posttraumatic OA model. Knee injury in the patellar groove was induced by microsurgery, as described previously (24). All animal procedures were performed according to institutional guidelines approved by the Home Office.

### Imaging accumulation of fluorescence-labeled 1-11E in the arthritic paw

An Alexa Fluor 680 protein labeling kit (Invitrogen, Paisley, UK) was used to label 1-11E and control anti-HEL, according to the manufacturer's instructions. Mice with AIA were rechallenged with mBSA 3 days before intravenous injection of an equal amount of fluorescent scFv (5 μg and 6.7 μg for 1-11E and anti-HEL, respectively). At various time points, the localization of the scFv was determined by placing mice anesthetized with isoflurane into an IVIS 100 Series imager (Caliper Life Sciences, Hopkinton, MA). High-resolution images were obtained by 2-second exposure using the Cy5 setting in the Living Image software (Caliper Life Sciences). In addition, the images were corrected for autofluorescence of the mice and the imaging box.

### Fusing 1-11E and anti-HEL scFv to murine tumor necrosis factor receptor II–Fc fusion protein (mTNFRII-Fc)

Polymerase chain reaction–amplified mTNFRII-Fc was cloned into pFastBac1.AH, which was created from pFastBac1 (Invitrogen), and fused to 1-11E or control anti-HEL scFv via the MMP-1 cleavage site (25). The construct was transformed into DH10Bac competent cells to generate bacmid vectors, followed by transfection of bacmid DNA into Sf9 insect cells using Cellfectin, according to the manufacturer's instructions (Invitrogen). Amplified virus was used to infect High Five insect cells for 72 hours for expression of the recombinant protein. Expression was analyzed by Western blotting using mouse anti–Tetra-His antibody (1:500 dilution; Qiagen, Crawley, UK) and HRP-conjugated anti-mouse antibodies (1:1,000 dilution; Sigma, Dorset, UK). Protein purification was performed using a nickel chelate column purification kit, according to the manufacturer's instructions (Qiagen). Overnight digestion of MMP-1 at 37°C was performed as described previously (25).

### Targeted delivery of TNFRII to arthritic joints

C57BL/6 mice with chronic-phase AIA were used. Mice were rechallenged with mBSA by injecting 50 μg mBSA in PBS into the knee of each animal 1 day before treatment. On days 1 and 3 after mBSA rechallenge, animals were injected intraperitoneally with 20 μg of 1-11E/mTNFRII-Fc or control HEL/mTNFRII-Fc, as described previously (26). When control mTNFRII-Fc (lacking the scFv, and thus with a molecular weight ∼50 kd lower) was used, we injected 13.33 μg of protein so that the molar concentrations of the various recombinant proteins were equivalent. Swelling of the knee was measured daily using calipers.

### Statistical analysis

Statistical analyses were undertaken using 1-tailed unpaired Mann-Whitney U test or repeated-measures analysis of variance, as applicable. An α value of 0.05 was used as the threshold for significance.

## RESULTS

### Generation of anti–modified CII scFv raised by the phage display human antibody library

**Figure 1:**
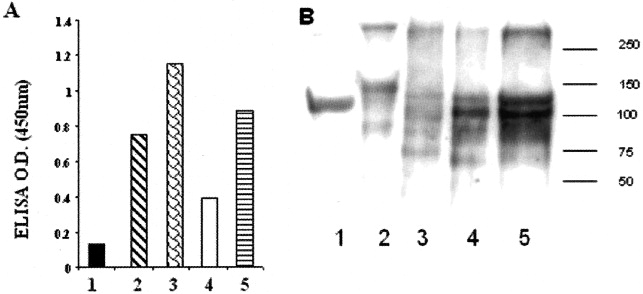
Binding of 1-11E to reactive oxygen species (ROS)–modified type II collagen (CII). **A,** An enzyme-linked immunosorbent assay (ELISA) to test binding of the anti–ROS-modified CII single-chain variable fragment (scFv) 1-11E to native and modified CII was performed as described previously (43). Briefly, a microtiter plate was coated with native CII or CII modified by glycation, HOCl, ^**.**^OH, or ONOO^−^. After blocking and incubation with 1-11E, mouse anti–Myc-Tag antibodies were added, followed by horseradish peroxidase (HRP)–conjugated anti-mouse antibodies, to probe bound scFv. In the ELISA, 1-11E bound to most types of modified CII (bars 2–5) but did not bind to native CII (bar 1). **B,** Western blotting was performed as described previously (16). Briefly, modified and native bovine CII were run on a sodium dodecyl sulfate gel under reducing conditions and then blotted onto a nitrocellulose membrane. After blocking, membranes were incubated with 1-11E, then with mouse anti–Myc-Tag, and then with HRP-conjugated anti-mouse antibodies. Binding of 1-11E to a range of CII α-chain fragments below 100 kd, as well as to aggregates with high molecular weight, was observed. Numbered horizontal bars represent the position of molecular weight markers, in kd. Lanes 1–5 represent native CII and CII modified by glycation, HOCl, ^**.**^OH, or ONOO^−^, respectively. OD = optical density.

After 3 rounds of subtractive selection, 42 phage clones with unique sequences specific to ROS-modified CII were selected. From among the most ROS-modified CII–specific clones that had no background binding to native CII, clone 1-11E had the best expression yield and became the focus of further studies. Most importantly, clone 1-11E exhibited binding to all forms of ROS-modified CII (Figure 1A). These findings suggested a capacity for identifying a wide range of potential oxidative modifications of CII by different ROS present during acute and chronic inflammation in arthritis. In addition, 1-11E did not bind to BSA modified by ROS (data not shown). Western blot analysis revealed that 1-11E bound to a range of fragments from the CII α-chain below 100 kd, as well as to aggregates of high molecular weight that result from the exposure of CII to ROS (16). Moreover, as we demonstrated previously for established RA sera (16), 1-11E also bound to the electrophoretic band that corresponds to the intact native CII α-chain polypeptide, although in an enzyme-linked immunosorbent assay, 1-11E did not bind to native CII (Figure 1B).

### Specific binding of 1-11E to damaged human articular cartilage

**Figure 2:**
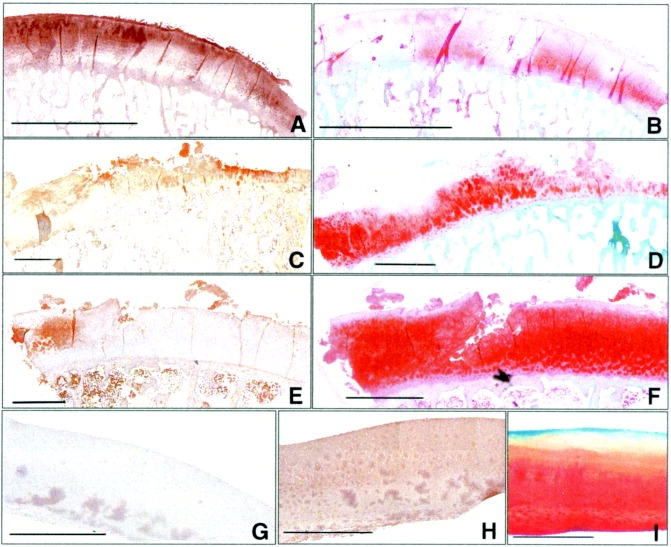
Binding of 1-11E to cartilage in patients with rheumatoid arthritis (RA) and osteoarthritis (OA). Antibody 1-11E diffusely stained RA cartilage in all layers (brown). **A,** Staining of the RA specimen in the superficial area and the middle zone was stronger than that in the deep zone. **B,** Staining of the RA specimen with Safranin O was weak and localized mainly in the deep zone (red). **C,** Staining of an OA cartilage sample with extensive erosions and marked surface damage, including the formation of fragments discrete from the parent cartilage, was strong in the most severely damaged area. **D,** Staining with 1-11E colocalized with an area of weak Safranin O staining in a parallel nonconsecutive OA cartilage section. **E** and **F,** Antibody 1-11E staining of cartilage from a patient with mild OA with typical fissuring of the surface of the upper cartilage appeared as a territorial “halo” around the chondrocytes (**E**), while staining with Safranin O was strong (**F**). **G–I,** Staining of the subchondral bone was not observed in any of the samples tested. No staining with 1-11E was detected in histologically normal cartilage (**G**), which also stained normally with a commercial anti–type II collagen monoclonal antibody (**H**) and with Safranin O (**I**). Bars in **A** and **B** = 5,000 μm; bars in **C–H** = 2,000 μm; bar in **I** = 500 μm.

The cartilage extracellular matrix is a complex structure where several molecules interact to form structural and functional units. Selected in vitro against purified ROS-modified CII, 1-11E may not recognize the tertiary and quaternary structure of collagens in the intact tissue. To determine binding specificity in the intact tissue, we tested the capacity of 1-11E to bind to ROS-modified CII within the cartilage matrix of arthritic cartilage from patients with RA, where inflammation is extensive, compared with that in OA, where inflammation is milder. As shown in Figure 2A, the staining of RA cartilage by 1-11E displayed a diffuse pattern in all layers of the section, reflecting the presence of high levels of ROS across the cartilage, which may result from the high influx of reactive immune cells into the joint. Staining in the deep zone, however, was weak. This staining pattern was opposite that of Safranin O, which showed strong staining in the deep zone, but weak or absent staining on the articular surface and in the middle zone (Figure 2B).

We next tested the binding of 1-11E to damaged cartilage from patients with OA, in whom the features of inflammation are less marked than those in patients with RA. Accordingly, in OA cartilage, strong staining was associated with cartilage areas exhibiting features of active OA, including altered Safranin O staining, cell clustering, and clefts. In an OA cartilage specimen with extensive erosions, strong staining was observed in the regions of severely damaged cartilage, with typical strong staining in areas of the fragmented cartilage (Figure 2C). Staining with Safranin O showed a pattern opposite that of 1-11E, with little overlap between the two (Figure 2D). In a specimen from a patient with mild OA with typical fissuring of the upper surface of the cartilage, immunostaining with 1-11E was specific to the damaged superficial layer, and there was strong staining as a territorial “halo” around the chondrocyte clusters (Figure 2E). This specimen stained strongly with Safranin O, as shown in Figure 2F, but exhibited weaker staining in the more severely damaged area (e.g., the cleft).

In all specimens, no staining with 1-11E was observed in the subchondral bone. Moreover, no staining with 1-11E was observed in histologically normal human cartilage (Figure 2G). However, strong and specific staining was observed when commercial anti-CII antibodies were used (Figure 2H), confirming that the lack of staining with 1-11E was not due to a lack of accessibility of CII in the intact undamaged cartilage. Hence, a canonical staining pattern with Safranin O was observed (Figure 2I).

### Staining of cartilage from mice with experimental arthritis

**Figure 3:**
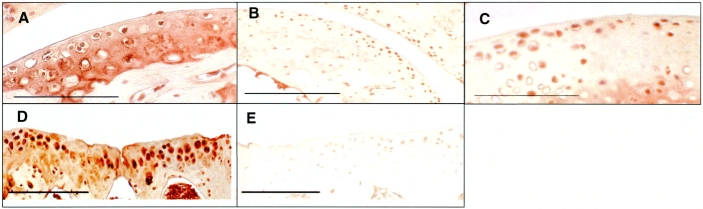
Binding of 1-11E to cartilage from mice with experimental inflammatory arthritis and posttraumatic osteoarthritis (OA). **A** and **B**, Inflamed cartilage was uniformly and strongly stained with 1-11E (**A**), while no staining was observed using irrelevant anti–hen egg lysozyme (anti-HEL) single-chain variable fragment (scFv) (**B**). **C,** Antibody 1-11E did not stain control uninflamed cartilage. **D,** Strong pericellular staining with 1-11E was observed in the damaged area of cartilage from mice with knee injury that developed posttraumatic OA. **E,** No binding was observed using control anti-HEL scFv. Bars = 100 μm.

To validate the findings in human RA cartilage sections, we studied cartilage from mice with experimental inflammatory arthritis (22). As seen in Figure 3A, cartilage from inflamed paws was strongly and uniformly stained with 1-11E but not with the irrelevant anti-HEL scFv (Figure 3B). In addition, 1-11E did not stain uninflamed preserved mouse cartilage (Figure 3C).

OA cartilage was obtained from C57BL/6 mice with knee injuries, which repair poorly and thus develop features of posttraumatic OA (24). Clear pericellular staining was observed in the damaged area stained with 1-11E (Figure 3D), while no staining was detected when staining with the control anti-HEL scFv (Figure 3E).

### Selective accumulation of 1-11E at the site of the inflamed paw

**Figure 4:**
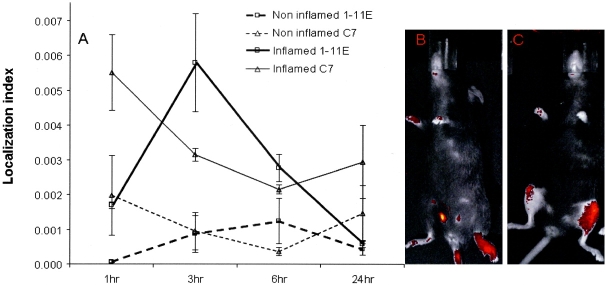
Selective accumulation of 1-11E in the inflamed paw. Mice with antigen-induced arthritis and similar degrees of paw swelling were injected intravenously with Alexa Fluor 680–labeled 1-11E and with control anti–hen egg lysozyme (anti-HEL [C7]) (n = 3 per group). Single-chain variable fragment (scFv) localization analysis was performed using ImageJ software (NIH Image, National Institutes of Health, Bethesda, MD; online at http://rsbweb.nih.gov/ij/); the RGB Split and Image Calculator functions in ImageJ were used to subtract background from signal. The localization index is the product of the area and the mean pixel density, corrected for scale bar variations between images (Excel; Microsoft, Redmond, WA). **A,** Greater accumulation of 1-11E was observed in inflamed paws than in uninflamed paws after 3 hours. Control anti-HEL scFv exhibited no specific accumulation, and the signal was reduced down to a constant level throughout the experiment. At 1 hour, the level of localized fluorescence-labeled 1-11E in inflamed paws was ∼3 times lower than the level of fluorescence-labeled anti-HEL (*P* = 0.05) in inflamed paws. At 3 hours, however, the level of localized fluorescence-labeled 1-11E was ∼2 times higher than the level of fluorescence-labeled anti-HEL in inflamed paws (*P* = 0.05). Values are the mean ± SD. **B** and **C,** Scans of mice with an inflamed left paw (**B**) or knee (**C**) that were injected with fluorescence-labeled 1-11E revealed specific accumulation of 1-11E in the inflamed tissue.

Next, we established whether anti–ROS-modified CII scFv would accumulate in vivo in the inflamed joint versus uninflamed joints following systemic administration during the chronic phase of the AIA model. Upon challenging animals with mBSA 3 weeks after the first stimulation, mice with a similar degree of paw swelling (n = 3) were injected intravenously with 1-11E labeled using an Alexa Fluor 680 protein labeling kit and with control anti-HEL. As shown in Figure 4A, by imaging the mice, we observed a greater accumulation of 1-11E in the inflamed paw, with maximum accumulation of 1-11E observed 3 hours after injection (*P* < 0.001). In contrast, irrelevant anti-HEL scFv showed no specific accumulation. The input fluorescence signal of anti-HEL scFv in the inflamed paw was reduced ∼40% at 3 hours and maintained this approximate level for the remainder of the experiment (Figure 4A). Moreover, accumulation of 1-11E was specific to the inflamed paw (Figure 4B) or knee (Figure 4C), with very low or no background localization in the uninflamed joints or other cartilaginous organs.

### Treatment of mice with AIA with an 1-11E and mTNFRII-Fc fusion protein product

**Figure 5:**
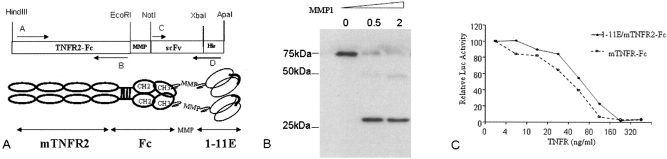
Fusion of 1-11E to soluble murine tumor necrosis factor receptor II–Fc fusion protein (mTNFRII-Fc). **A,** Schematic representation of the 1-11E/mTNFRII-Fc construct containing a matrix metalloproteinase (MMP) cleavage site between 1-11E and mTNFRII-Fc. Murine TNFRII-Fc was amplified with forward primer A (5′-GCTAAGCTTATGGCGCCCGCCGCCCTC) and reverse primer B (5′-CTTGAATTCTTTACCCAGAGACCGGGA). After digestion, the polymerase chain reaction (PCR)–amplified fragment was cloned into the *Hind* III–*Eco* RI sites of pFastBac1.AH, which was created from pFastBac1 (Invitrogen) containing an MMP-1 cleavage site cloned between *Eco* RI and *Not* I. Antibody 1-11E or control anti–hen egg lysozyme (anti-HEL) single-chain variable fragment (scFv) was amplified with forward primer C (5′-CAGGCGGCCGCAATGGCCGAGGTGCAGCTG-3′) and reverse primer D (5′-CTTGGGCCCTCAATGGTGGTGGTGATGGTGTCTAGACCGTTTGATTTCCACCTT-3′) to amplify the scFv and to include a His-Tag between *Xba* I and *Apa* I. The PCR-amplified fragment was then digested and cloned into pFastBac1.AH digested with *Not* I and *Apa* I. The fusion protein was expressed using the baculovirus expression system. **B,** Western blot analysis of the fusion protein product with the expected molecular weight band of 75 kd, which was reduced to 25 kd after cleavage with MMP-1, corresponding to the scFv detected by the anti–His-Tag. **C,** Measurement of the activity of the 1-11E/mTNFRII-Fc fusion protein. The activity of the 1-11E/mTNFRII-Fc fusion protein was similar to that observed with mTNFRII-Fc alone, as measured by inhibition of TNFα-mediated induction of the NF-κB promoter–driven luciferase reporter gene (luc) in HeLa 57A cells. Values are the mean.

To investigate the ability of 1-11E to deliver soluble TNFRII to the arthritic joint, 1-11E was fused to mTNFRII-Fc. In addition, an MMP cleavage site was inserted to assure the release of mTNFRII-Fc at the site of the inflammation (25) (Figure 5A). Western blot analysis revealed the fusion protein product with the expected ∼75 kd molecular weight band, which reflects the total of the predicted ∼50 kd mTNFRII-Fc combined with the ∼25 kd scFv. Incubation of the fusion protein with recombinant MMP-1 resulted in detection of a band in the region of 25 kd corresponding to the scFv cleaved from mTNFRII-Fc (Figure 5B). The biologic activity of mTNFRII-Fc was then tested by determining its ability to inhibit in vitro the TNFα-mediated activation of a reporter luciferase gene driven by an NF-κB promoter. From this, it was concluded that the potency of the fusion of soluble 1-11E with mTNFRII-Fc as an inhibitor of TNFα was as strong as soluble TNFRII-Fc alone (Figure 5C).

**Figure 6:**
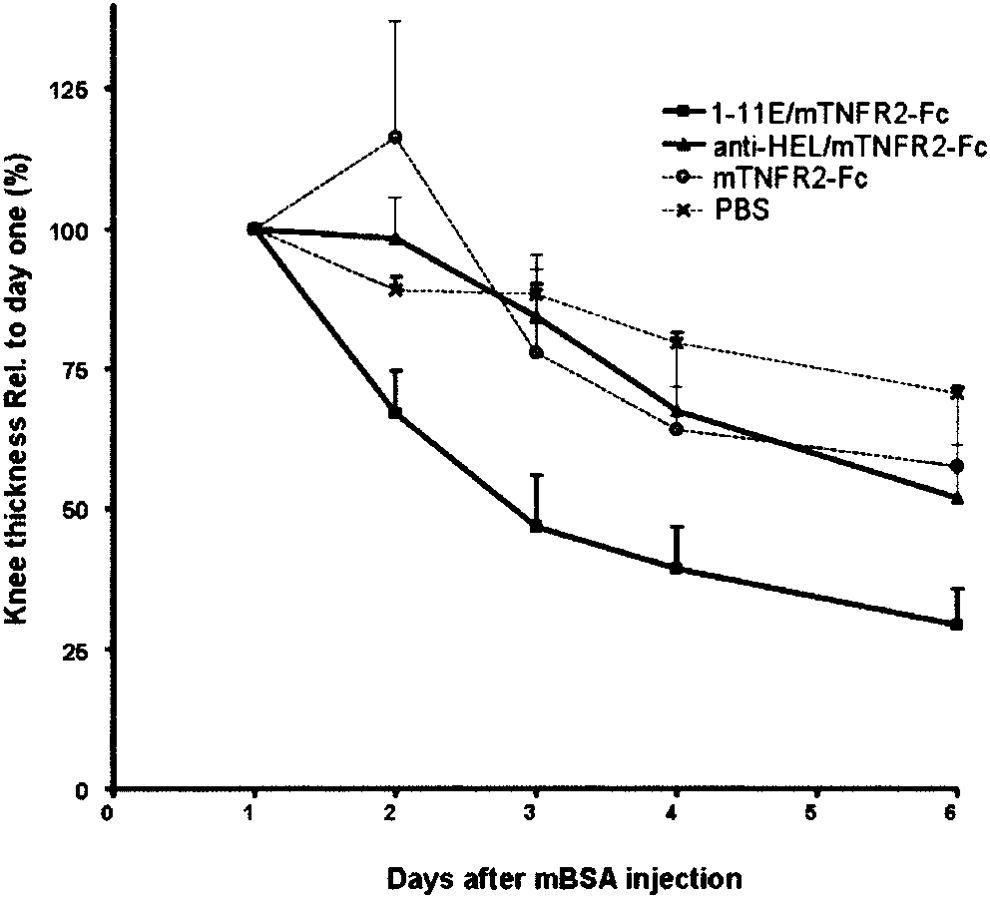
Superior therapeutic effect of 1-11E/murine tumor necrosis factor receptor II–Fc fusion protein (mTNFRII-Fc) in the antigen-induced arthritis (AIA) model. We used mice with AIA during the chronic phase of disease that were rechallenged with methylated bovine serum albumin (mBSA), followed by injection of therapeutic protein on day 1 and day 3 (n = 8 for the 1-11E/mTNFRII-Fc and the hen egg lysozyme [HEL]/mTNFRII-Fc treatment groups; n = 7 for the mTNFRII-Fc and phosphate buffered saline [PBS] control groups). Reduction of swelling of inflamed knees was accelerated in mice in the 1-11E/mTNFRII-Fc fusion group as compared with the control anti-HEL/mTNFRII-Fc and mTNFRII-Fc groups (*P* = 0.0008 by repeated-measures analysis of variance). *P* values for the post hoc test were calculated using the Newman-Keuls multiple comparison test: for 1-11E/mTNFRII-Fc versus HEL/mTNFRII-Fc, *P* < 0.001; for 1-11E/mTNFRII-Fc versus mTNFRII-Fc, *P* < 0.01; and for HEL/mTNFRII-Fc versus mTNFRII-Fc, *P* > 0.05. These results indicate that 1-11E specifically accelerates the reduction of knee swelling. Values are the mean and SD.

The therapeutic potency of the fusion of 1-11E and mTNFRII-Fc was tested in experimental arthritis using the chronic AIA protocol. After rechallenge with mBSA, mice with similar degrees of knee swelling were selected for treatment. Intraperitoneal injection of 1-11E fused with mTNFRII-Fc on day 1 and day 3 after rechallenge significantly reduced knee swelling compared with the administration of either control anti-HEL fused with mTNFRII-Fc (*P* < 0.001) or control mTNFRII-Fc alone (*P* < 0.01) (Figure 6).

## DISCUSSION

The advent of biologic DMARDs such as anti-TNFα has revolutionized the treatment of RA. However, systemic administration does not deliver pharmacologically active molecules solely to arthritic joints and therefore could contribute to side effects. Targeted therapy would concentrate the bioactive molecules within the damaged joints and, thus, could increase potency while minimizing side effects. However, a major problem with applying targeted therapies in arthritis has been the identification of specific markers of inflamed joint tissue to deliver the antiinflammatory agent. An scFv specific to a marker of angiogenesis was previously used to target cytokines in mice with collagen-induced arthritis (CIA) (27). An angiogenic marker, however, is not specific to arthritic joint tissue, and so the problem has remained unresolved. We hypothesized that CII is the best candidate for targeting therapy to the joint because it is specific to cartilage. Nevertheless, there is a need to find a way to target the drugs solely to the damaged joints, since CII is a major component of both healthy and arthritic joints. To allow targeting to a damaged joint independently of the etiology of the damage, we hypothesized that CII modified by ROS, which is present only in the damaged arthritic joint, would be a suitable target.

Using a phage display human antibody library, we developed a panel of human scFv that bind only to CII modified in vitro by known reactive oxidants in RA (28,29). Out of many clones tested, clone 1-11E bound to all forms of ROS-modified CII and had the capacity to recognize a wide range of potential oxidative modifications of CII by different ROS present during acute and chronic inflammation. Similar to the findings in serum samples from patients with established RA, 1-11E bound to a range of fragmented and aggregated CII bands as well as to native CII, as determined by Western blotting (16,28–32). The binding of 1-11E to native CII was probably 1-11E binding to denatured epitopes of native CII, which result from the strong denaturing conditions in sodium dodecyl sulfate gels. In addition, some CII oxidation could occur during gel electrophoresis.

Most importantly, 1-11E bound only to arthritic cartilage and not to normal intact cartilage. We analyzed the binding of 1-11E to damaged cartilage in 2 types of arthritis, RA and OA. The staining pattern observed in RA cartilage was fundamentally different from that observed in OA cartilage, reflecting the basic difference between RA and OA pathogenesis. Strong, diffused staining of RA cartilage with 1-11E was observed. Staining of the deep zone, however, was very weak. Interestingly, staining with Safranin O (which stains sulfated, negatively charged glycosaminoglycans and is thus directly proportional to the intact proteoglycan content) was detected mainly in the deep zone of the RA specimen. This reverse pattern may reflect a high influx of infiltrating immune cells within the synovial membrane, producing high levels of ROS that mostly modify both the superficial layers, which have direct contact with the inflamed synovial fluid and the synovial membrane pannus, and the middle zone, which is more accessible than the deep zone. When cartilage from mice with AIA, an experimental model of inflamed arthritis, was stained with 1-11E, it exhibited strong diffused staining similar to that observed in human RA cartilage.

Synovial inflammation in OA is not as extensive as in RA, and mediators of inflammation appear to be produced mainly by chondrocytes (13,15). The inflammatory and catabolic events leading to cartilage loss in RA and OA are different entities and have different origins. In RA, mediators of inflammation originate from the inflamed synovial membrane and are extrinsic to cartilage, whereas in OA, they originate from the chondrocytes themselves. This may explain the strikingly different patterns we observed with 1-11E staining, which was diffuse in RA samples and pericellular or territorial in OA samples. Although 1-11E stained degraded OA cartilage, it is interesting that 1-11E staining did not always correlate with certain features of OA, such as decreased staining with Safranin O, structural features (clefts), and cellular features (clusters and hypocellularity). Such features reflect the cumulative damage to cartilage and the final balance between degradation and anabolism, whereas staining with 1-11E reflects a snapshot of local ROS-mediated collagen modification/inflammation.

For example, in the cartilage sample from a patient with mild OA, the territorial and pericellular 1-11E staining around the chondrocyte clusters implies that there is enhanced inflammatory activity and high levels of ROS-modified CII, overlapping with strong staining with Safranin O, which may reflect enhanced anabolic activity (33). This may be an important feature of 1-11E staining in diseases such as OA, which have alternating periods of progression and quiescence or even partial recovery (34). Such a feature of 1-11E may also help to identify patients in whom cartilage degradation is predominantly driven by inflammation rather than by a lack of anabolic activity. Interestingly, in the cartilage sections from mice with experimental posttraumatic OA, we observed pericellular staining with 1-11E, which was similar to the staining with antibodies specific to neoepitopes generated by MMP-cleaved CII, as described previously (24). A previous correlation between oxidation and OA cartilage degradation was demonstrated by Yudoh et al (35), who observed strong staining for nitrotyrosine and a low antioxidant capacity in the degenerative region of OA cartilage compared with the intact region from the same sample. In conclusion, the different staining pattern produced by 1-11E in RA cartilage versus OA cartilage may reflect the differences between RA, in which inflammation is driven by synovitis, and OA, in which inflammation is predominantly driven by chondrocytes (13,15).

The inflamed joint and cartilage represent a complex structure, and there was a possibility that 1-11E would not be able to access ROS-modified CII in the inflamed joint in vivo. After intravenous injection of 1-11E into mice with AIA, we observed a specific accumulation of 1-11E in the inflamed paw, with maximum accumulation 3 hours after the injection, consistent with the known 2-hour half-life of the scFv (36). In contrast, irrelevant anti-HEL scFv exhibited no specific accumulation, but rather, it exhibited nonspecific localization due to increased extravasation into the inflamed joint versus the uninflamed joint.

The potential of 1-11E to facilitate the targeting of therapeutics specifically to the inflamed joint was assessed by fusing 1-11E to soluble mTNFRII as a proof of concept. A therapeutic effect was observed for 1-11E/mTNFRII-Fc, since it accelerated the reduction of inflamed knee swelling, compared with the effects of irrelevant anti-HEL/mTNFRII-Fc and control mTNFRII-Fc. We have therefore clearly demonstrated for the first time specific targeting of a therapeutic agent to inflamed joints by using damaged, cartilage-specific epitopes present specifically in the inflamed joints. Although CII is a major component of both healthy and arthritic joints, an antibody specific to ROS-modified CII, 1-11E, targeted a therapeutic moiety (mTNFRII-Fc) solely to the damaged arthritic joint, because ROS-modified CII is present only in inflamed joints. Interestingly, while 1-11E is cleared from the circulation in ∼3–6 hours, the therapeutic effect of 1-11E/mTNFRII-Fc lasted for a few days. This is consistent with previous observations in mice with experimental CIA that were injected with mTNFR-Fc (1,37,38). The longer therapeutic effect reflects subsequent changes in downstream biologic events that are hierarchically controlled by TNFα (1) and are therefore blocked as a result of TNFα blockade. In addition, we anticipate that the longer serum half-life of anti–ROS-modified CII/mTNFRII-Fc (molecular weight ∼150 kd versus ∼27 kd for the anti–ROS-modified CII scFv) may have contributed to longer efficacy (39).

We believe that this development, which would allow for the delivery of drugs specifically to inflamed joints, has the potential to revolutionize the treatment of RA, although further studies are needed to optimize the pharmacokinetics of this scFv. The development may also have a significant impact on the treatment of OA, which has lagged well behind that of RA (40,41). Once optimized for the clinical setting (and depending on the size of the payload drug), 1-11E, as an scFv, offers a small drug-delivery system with increased tissue penetration, short systemic half-life, and renal clearance in vivo (39). It has recently been demonstrated that the anti-TNFα scFv, ESBA 105, had superior synovial and cartilage penetration over intact infliximab, using a short-term monarthritis model in which rats were injected intraarticularly with an anti-TNFα scFv (42). Antibody 1-11E offers an advantage over the anti-TNFα scFv, since it will specifically deliver the anti-TNF therapy to the affected joint, even when applied systemically, without the need for intraarticular injection. In fact, once optimized for clinical use, 1-11E could have a dramatic impact on treatment efficacy and modality. A drug could be injected using the standard systemic administration, such as intravenous injection, but with a significant increase in potency and with minimized side effects.

In summary, regardless of the disease pathology, whether OA or RA, 1-11E has the potential for targeting anti-TNFα, other inflammatory cytokine blockers, or cartilage regenerating factors specifically to diseased tissues.

## AUTHOR CONTRIBUTIONS

All authors were involved in drafting the article or revising it critically for important intellectual content, and all authors approved the final version to be published. Dr. Nissim had full access to all of the data in the study and takes responsibility for the integrity of the data and the accuracy of the data analysis.

**Study conception and design.** Hughes, Faurholm, Dell'Accio, Chernajovsky, Nissim.

**Acquisition of data.** Hughes, Faurholm, Dell'Accio, Manzo, Seed, Eltawil, Marrelli, Gould, Subang, Nissim.

**Analysis and interpretation of data.** Hughes, Faurholm, Dell'Accio, Seed, Al-Kashi, De Bari, Winyard, Chernajovsky, Nissim.
